# Supplementary material for: IAP retrotransposons contribute to the transcriptional diversity of the murine placenta
Source: Life Sci Alliance. 2026 Nov 4;10(1):e202603923. doi: 10.26508/lsa.202603923 (PMC13736139; doi:10.26508/lsa.202603923)
Supplement: Supplementary file 1 [file LSA-2026-03923_review_history.pdf]

IAP retrotransposons contribute to the transcriptional diversity of the murine placenta

Samuele Amante, Maria Vignola, Cyril Pulver, Tessa Bertozzi, Anne Ferguson-Smith, Marika Charalambous, and Miguel Branco
DOI: https://doi.org/10.26508/lsa.202603923

Corresponding author(s): Miguel Branco, Queen Mary University of London and Samuele Amante, Queen Mary University of London

Review Timeline:

Submission Date:	2026-09-30
Editorial Decision:	2026-10-16
Revision Received:	2026-10-18
Accepted:	2026-10-20

Scientific Editor: Tim Fessenden

Transaction Report:

(Note: With the exception of the correction of typographical or spelling errors that could be a source of ambiguity, letters and reports are not edited. The original formatting of letters and referee reports may not be reflected in this compilation.)

Please note that the manuscript was reviewed at *Review Commons* and these reports were taken into account in the decision-making process at ***Life Science Alliance.***

Reviews

Review #1

1. Evidence, reproducibility and clarity:

In this report the authors have provided evidence for the involvement of transposable elements in the regulation of gene expression in murine trophoblast cells and placenta. They utilized data that they generated and also published data in their analyses. They concluded that the involvement of transposable elements in the regulation of genes in differentiated trophoblast cells was less than utilized in trophoblast cells in the stem state. They provide evidence for the utilization of intracisternal A particle elements in the modulation of gene transcription of the mouse placenta, which represents a more recent evolutionary adaptation. Overall the report is descriptive presenting correlations with limited testing of specific hypotheses. There is also the impression that the manuscript consists of the merging of two projects, which have not been fully developed. Some concerns with the experimental design and interpretation of the results are provided below.

1. Some concerns with the model systems used in the analysis. First of all, there are methods for inducing the differentiation of mouse trophoblast stem cells, which usually involves the removal of factors that promote trophoblast stem cell proliferation. The authors do not describe their method for inducing trophoblast stem cell differentiation nor did they show evidence that they directly investigated differentiated trophoblast stem cells.
2. There is a published report presenting data from single cell analysis of mouse trophoblast stem cells in the stem and differentiated states that was not acknowledged or used in the authors' analyses. Please see: Angelova et al. 2025 Nature Communications (PMID:39747179).
3. Much of the analysis, compared mouse trophoblast stem cells and murine placentas. Interpretation of single nucleus sequencing data from mouse placentas can provide information regarding the behavior of trophoblast cells; however, bulk sequencing of placentas is limited. The placenta contains trophoblast cell and non-trophoblast cell components. More specifically the placenta contains fetal endothelial, immune, and mesenchymal cells and depending upon dissections and the gestational stage of dissections variable amounts of uterine decidua and yolk sac-derived tissues. The authors need to be clear in the comparisons that they are making. More specifically, the authors need to effectively communicate the cell types from the placenta contributing to the results they are describing. Attributing analyses of the placenta to trophoblast cells is problematic.
4. How were the newly derived mouse trophoblast stem cells characterized? Do they behave like authentic mouse trophoblast stem cells? Were the newly derived trophoblast stem cells capable undergoing differentiation? What parameters were measured?
5. Were analyses with the newly derived mouse trophoblast stem cells performed in the stem or differentiated states?
6. TSC derivation and culture section. The authors appear to be initially describing the generation of mouse embryonic fibroblast conditioned medium not trophoblast stem cell conditioned medium as stated. Some clarification will be helpful. As stated above, the authors do not provide any information on the characterization and validation of the newly derived mouse trophoblast stem cells, which is problematic.
7. Discussion. The authors state that there are fundamental differences between the mouse and human placenta regarding the co-option of transposable element subfamilies. Human trophoblast stem cells represent a highly tractable model and could be compared with mouse trophoblast stem cells to further explore this observation.

2. Significance:

Efforts to understand roles for transposable elements in the regulation of trophoblast cell gene expression and placental evolution are very important. We recognize significant differences in placentation across various species but do not have a good understanding how this important developmental process evolved.

Assessment: The authors have a potentially interesting story. However, it appears that they have merged two incomplete research efforts: i) effects of trophoblast cell differentiation on utilization transposable elements to regulate gene expression; ii) IAP involvement in regulating murine placental transcription.

Advance: The scientific advance is somewhat fragmented. There is a reinforcement of our existing understanding of the involvement of transposable elements in trophoblast and placental gene regulation but other new insights are limited or not well developed.

Audience: Evolutionary biologists and reproductive and developmental biologists.

3. How much time do you estimate the authors will need to complete the suggested revisions:

Cannot tell / Not applicable

4. *Review Commons* values the work of reviewers and encourages them to get credit for their work. Select 'Yes' below to register your reviewing activity at Web of Science Reviewer Recognition Service (formerly Publons); note that the content of your review will not be visible on Web of Science.

Web of Science Reviewer Recognition

No

Review #2

1. Evidence, reproducibility and clarity:

Summary: This manuscript describes the characterization of transposable elements (TEs) in mouse trophoblast stem cells and in the mature mouse placenta. The authors find that overall, the trophoblast stem or progenitor state of TSCs harbours a greater abundance of active TE elements, while their activity levels decline as trophoblast differentiates. Instead, the dominant repetitive element that is active in the mature placenta are intracisternal A particle (IAP)-derived elements. Indeed, the authors show that these provide the initiation sites for differential isoforms of some 27 chimeric transcripts that are specific to differentiated trophoblast cell types. The authors attempt to epigenetically silence these IAPs in TSCs using CRISPRi methodolgy, and find reduced expression of 4 IAP-driven transcripts and many presumably secondary transcriptional changes. Finally, they also compare IAP activity in the placentas of different mouse species or sub-species, and conclude that IAP insertion sites close to genes can affect their expression in the placenta, with potential consequences for development and evolution.

Major comments:

This is a well-conducted study that brings significant novelty, albeit to a more specialized audience.

There are several aspects that need clarification, addition and some experimental work:

1. Figure 1A shows carefully separated cell types, in particular extraembryonic mesoderm, that have also been assessed by the various cut&tag and ATAC-seq methods, but are not mentioned in the remainder of the manuscript. This should be added. I.e., is the same activity pattern of IAPs evident in the ExMes cells, or do they follow a more somatic pattern?
2. Page 4, top: The mention of a "custom pipeline" for cut&tag analysis is vague, and the modifications and what they stand for is hardly mentioned. These details need to be elaborated, so to be more accessible to a wider audience.
3. For differentiated TSCs, only ATAC-seq data were analysed. How do they relate to the various cut&tag profiles, and do they result in a robust detection of putative active repeat elements at a detection limit similar to the chromatin marks? I would think that it might be prudent to include the same cut&tag for differentiated TSCs as well, so to be directly comparable to the other data. This is important to establish whether TE elements are really less active in differentiating trophoblast, or whether this feature is intrinsic to the placenta and not to pure trophoblast cells in culture, in which case it may be influenced by tissue context.
4. Are the IAP-initiated chimeric transcripts including new coding regions? If so, a Western Blot analysis of a few of the 27 candidates should be performed to prove this. Suv39h2 is a particularly interesting candidate where such protein analysis would be very informative.
5. A WB analysis should for sure be performed on the M. musculus and M. pahari placentas. The IHC staining is not interpretable as to whether or not SUV39H2 levels are reduced in M pahari.
6. Could the authors please also provide more global proof of the CRISPRi success. The display of two candidate gene tracks is not very telling.

2. Significance:

General assessment:

Collectively, this is a carefully conducted study that needs to be bolstered by some few additional experiments, as suggested above. The discovery of changing patterns of repetitive element activity in differentiating trophoblast cells is important and intriguing, as it has direct impact on the evolutionary divergence of gene expression and, as a consequence, cell type differentiation, through the insertion of IAP and L1 elements close to placenta-expressed

genes. This will be a major contributor and even driver of the barrier to inter-species hybridization that the placenta represents.

Advance:

Currently, the main TE elements known to drive placenta-specific gene expression are retrovirally derived LTR elements. Here, however, the authors show that the relevance of these elements diminishes in the mature placenta, and instead is taken over by a different class, the IAP elements. This is important, as many these elements retain the capacity for retrotransposition, and thus actively contribute to ongoing evolutionary divergence of placental gene expression patterns that ultimately may drive speciation.

Audience:

The manuscript is not particularly easy to follow, even for the informed reader, and it appeals to a relatively specialized audience in the field of genome regulation coupled to evolutionary aspects of repetitive element insertion/transposition. The authors should be encouraged to spell out some aspects of their thought process throughout the study in some more detail, so not to "lose" the reader.
The study would sit well in journals that cover a wide spectrum of biology.

3. How much time do you estimate the authors will need to complete the suggested revisions:

Between 3 and 6 months

4. *Review Commons* values the work of reviewers and encourages them to get credit for their work. Select 'Yes' below to register your reviewing activity at Web of Science Reviewer Recognition Service (formerly Publons); note that the content of your review will not be visible on Web of Science.

Web of Science Reviewer Recognition

No

Review #3

1. Evidence, reproducibility and clarity:

The cis-regulatory roles of TEs in human/mouse TSCs have been extensively studied, yet in vivo studies on their roles in placenta tissue is largely absent. In this manuscript, Amante and colleagues compared the regulatory landscape of TEs across the trophoblast cell lines and placenta samples in human and mouse, and after revealing the shared and species-specific patterns (including some that are surprising), they further investigated the regulatory function of the murine-specific IAP retrotransposons in house mouse and other mouse strains. Specifically, it presents several findings regarding the shared and diverged function of TEs across: 1) in vivo vs. in vitro placental models, 2) human vs. mouse, 3) and different mouse strains. The writing is of good quality, the results are well visualized and interpreted, the conclusions are reasonable, and the novelty is high. It significantly extended previous studies from the same group as well as many other researchers. I think this manuscript should fit publication after a minor revision. Below I have a few comments:

1. In Fig. 1B, it seems the differences of TE enrichment between the same groups of samples (e.g., B6 TSC vs. GFP TSC) is also remarkable. Is this expectable? I am curious if such difference is robust, or it is just due to the TE sub-families with too few copies, whose enrichment can be influenced by just a couple of overlapping counts. The authors may double-check if possible.
2. The authors demonstrate that the association of TEs to cis-regulatory elements is much weaker in the placenta of mouse relative to human, and in mouse the activation of TEs is indeed similar to most other tissues. And based on this observation, they propose that "Co-option of TEs as regulatory elements within the mature placenta may therefore not be as promiscuous across species as commonly thought" (page 4 paragraph 1). While this finding is quite interesting, how it is related to the popular hypothesis that "maternal-fetal conflict leads to the strong TE activation in placenta"? I am curious if the authors have any idea on this point.
3. For the highly active IAP subfamilies identified in mouse placenta, have the authors tried to identity the enriched motifs, which may be helpful for uncovering transcription factors responsible for their activation?
4. In Fig. 3B, the IAPEY_LTR-adjacent Zfp229 gene is demonstrated, yet this gene is not mentioned at all in the main text. The authors may consider providing more details for this gene.
5. A few errors for the citations should be corrected. For example, the journal names are missed for ref56 and ref58 at

page 22.
6. A few typos should be corrected. For example, at page 11 line 2, "of" is missed between "presence this IAP-driven.

2. Significance:

Overall, this is an interesting and technically-sound study with substantial novelty, which significantly extends previous knowledge on TE function in placenta which largely relies on in vitro models.I believe this study will be attractive to the fields about TE function and placenta evolution.

3. How much time do you estimate the authors will need to complete the suggested revisions:

Less than 1 month

4. *Review Commons* values the work of reviewers and encourages them to get credit for their work. Select 'Yes' below to register your reviewing activity at Web of Science Reviewer Recognition Service (formerly Publons); note that the content of your review will not be visible on Web of Science.

Web of Science Reviewer Recognition

Yes

We sincerely thank the reviewers for the time dedicated to providing us with feedback on our work. In response to their helpful remarks, we have generated new data and made several changes to the manuscript. We hope the reviewers agree that, together with the rebuttal points below, this improved version addresses their concerns.

Reviewer #1

Evidence, reproducibility and clarity

In this report the authors have provided evidence for the involvement of transposable elements in the regulation of gene expression in murine trophoblast cells and placenta. They utilized data that they generated and also published data in their analyses. They concluded that the involvement of transposable elements in the regulation of genes in differentiated trophoblast cells was less than utilized in trophoblast cells in the stem state. They provide evidence for the utilization of intracisternal A particle elements in the modulation of gene transcription of the mouse placenta, which represents a more recent evolutionary adaptation. Overall the report is descriptive presenting correlations with limited testing of specific hypotheses. There is also the impression that the manuscript consists of the merging of two projects, which have not been fully developed. Some concerns with the experimental design and interpretation of the results are provided below.

1. Some concerns with the model systems used in the analysis. First of all, there are methods for inducing the differentiation of mouse trophoblast stem cells, which usually involves the removal of factors that promote trophoblast stem cell proliferation. The authors do not describe their method for inducing trophoblast stem cell differentiation nor did they show evidence that they directly investigated differentiated trophoblast stem cells.

We have added information in the Methods section to clarify that differentiation was performed by culturing cells in TS base medium (no conditioned media, FGF or heparin) for 4 days. We also provide RT-qPCR data confirming TSC differentiation (Figure S1A).

2. There is a published report presenting data from single cell analysis of mouse trophoblast stem cells in the stem and differentiated states that was not acknowledged or used in the authors' analyses. Please see: Angelova et al. 2025 Nature Communications (PMID:39747179).

We appreciate the reviewer’s suggestion, but the purpose of our single-cell analysis was to assess the expression of IAP elements and their associated chimeric transcripts in vivo. This has more significance than single-cell data from in vitro differentiated cells. We also found that at least some of the chimeric transcripts seen in vivo are not detected in vitro.

3. Much of the analysis, compared mouse trophoblast stem cells and murine placentas. Interpretation of single nucleus sequencing data from mouse placentas can provide information regarding the behavior of trophoblast cells; however, bulk sequencing of placentas is limited. The placenta contains trophoblast cell and non-trophoblast cell components. More specifically the placenta contains fetal endothelial, immune, and mesenchymal cells and depending upon dissections and the gestational stage of dissections variable amounts of uterine decidua and yolk sac-derived tissues. The authors need to be clear in the comparisons that they are making. More specifically, the authors need to effectively communicate the cell types from the placenta contributing to the results they are describing. Attributing analyses of the placenta to trophoblast cells is problematic.

We agree with this point. In our original submission we had included a cell type deconvolution analysis to infer the composition of our bulk placental tissue (Figure S1B of the revised submission). This clarifies the heterogeneity of the tissue and shows that most cells are trophoblast. We also used a genetic model to isolate trophoblast from placentas to address this point. Cell type deconvolution confirms that >90% of cells are trophoblast.

4. How were the newly derived mouse trophoblast stem cells characterized? Do they behave like authentic mouse trophoblast stem cells? Were the newly derived trophoblast stem cells capable undergoing differentiation? What parameters were measured?

We now include data on trophoblast stem cell and differentiation markers, comparing our newly derived line with the well-established GFP-TSC line (Figure S1A). While not included in this submission, the cells also presented with the expected morphologies when cultured under stem or differentiation conditions.

5. Were analyses with the newly derived mouse trophoblast stem cells performed in the stem or differentiated states?

Our initial analyses were only from cells cultured in stem conditions. However, we now also include an analysis of TE regulatory activity in differentiation conditions (updated Figures 1B, 1D, S1C).

6. TSC derivation and culture section. The authors appear to be initially describing the generation of mouse embryonic fibroblast conditioned medium not trophoblast stem cell conditioned medium as stated. Some clarification will be helpful. As stated above, the authors do not provide any information on the characterization and validation of the newly derived mouse trophoblast stem cells, which is problematic.

We appreciate the confusion with the nomenclature. To clarify we have changed the start of that section to: "Conditioned medium for the culture of TSCs (TS-CM) was prepared by…". This conditioned medium is generated using MEFs and is then used to culture TSCs.

7. Discussion. The authors state that there are fundamental differences between the mouse and human placenta regarding the co-option of transposable element subfamilies. Human trophoblast stem cells represent a highly tractable model and could be compared with mouse trophoblast stem cells to further explore this observation.

We previously published a paper focused on TE co-option in human trophoblast (PMID: 37012406), and we made a brief comparison to those data in the current manuscript (Figure S1G). Interestingly, in contrast to mouse, many of the TEs with regulatory activity in human TSCs remain active in term placenta.

Significance

Efforts to understand roles for transposable elements in the regulation of trophoblast cell gene expression and placental evolution are very important. We recognize significant differences in placentation across various species but do not have a good understanding how this important developmental process evolved.

Assessment: The authors have a potentially interesting story. However, it appears that they have merged two incomplete research efforts: i) effects of trophoblast cell differentiation on utilization transposable elements to regulate gene expression; ii) IAP involvement in regulating murine placental transcription.

Whilst we appreciate this viewpoint, our investigation of IAPs as regulators of gene expression was triggered from the analysis in the first part of the manuscript and thus follows logically in our view. Moreover, the overarching theme remains consistent: the effects of TEs (whether IAPs or others) on gene expression/transcription.

Advance: The scientific advance is somewhat fragmented. There is a reinforcement of our existing understanding of the involvement of transposable elements in trophoblast and placental gene regulation but other new insights are limited or not well developed.

Both the TE and placental scientific communities largely assume that the placenta is a privileged organ for co-option of TEs as regulatory elements. Here we demonstrate that TE co-option in the placenta can be quite limited and species-specific. Additionally, the roles of IAPs as gene regulators in the placenta had not been previously described. We believe these two novel

observations constitute significant advancements in the field.

Audience: Evolutionary biologists and reproductive and developmental biologists.

Reviewer #2

Evidence, reproducibility and clarity

Summary: This manuscript describes the characterization of transposable elements (TEs) in mouse trophoblast stem cells and in the mature mouse placenta. The authors find that overall, the trophoblast stem or progenitor state of TSCs harbours a greater abundance of active TE elements, while their activity levels decline as trophoblast differentiates. Instead, the dominant repetitive element that is active in the mature placenta are intracisternal A particle (IAP)-derived elements. Indeed, the authors show that these provide the initiation sites for differential isoforms of some 27 chimeric transcripts that are specific to differentiated trophoblast cell types. The authors attempt to epigenetically silence these IAPs in TSCs using CRISPRi methodolgy, and find reduced expression of 4 IAP-driven transcripts and many presumably secondary transcriptional changes. Finally, they also compare IAP activity in the placentas of different mouse species or sub-species, and conclude that IAP insertion sites close to genes can affect their expression in the placenta, with potential consequences for development and evolution.

Major comments:
This is a well-conducted study that brings significant novelty, albeit to a more specialized audience.

There are several aspects that need clarification, addition and some experimental work:
1. Figure 1A shows carefully separated cell types, in particular extraembryonic mesoderm, that have also been assessed by the various cut&tag and ATAC-seq methods, but are not mentioned in the remainder of the manuscript. This should be added. I.e., is the same activity pattern of IAPs evident in the ExMes cells, or do they follow a more somatic pattern?

Apologies if additional analyses of extraembryonic mesoderm were not obvious, but we did analyse IAP expression in these cells and show in Figure 2B that it is much lower when compared to trophoblast. We also used the comparison between trophoblast and extraembryonic mesoderm in Figures 2A, 3B, S2A-D and S4B.

2. Page 4, top: The mention of a "custom pipeline" for cut&tag analysis is vague, and the modifications and what they stand for is hardly mentioned. These details need to be elaborated, so to be more accessible to a wider audience.

We have tried to clarify the overall strategy of the analysis: "Using CUT&Tag and ATAC-seq data, we aimed to identify TE subfamilies that bear classic hallmarks of active promoters (open chromatin, H3K4me3, H3K27ac) and/or enhancers (open chromatin, H3K27ac, H3K4me1). We used a custom pipeline that selects TE subfamilies bearing more elements overlapping CUT&Tag/ATAC-seq peaks than expected by chance.".

3. For differentiated TSCs, only ATAC-seq data were analysed. How do they relate to the various cut&tag profiles, and do they result in a robust detection of putative active repeat elements at a detection limit similar to the chromatin marks? I would think that it might be prudent to include the same cut&tag for differentiated TSCs as well, so to be directly comparable to the other data. This is important to establish whether TE elements are really less active in differentiating trophoblast, or whether this feature is intrinsic to the placenta and not to pure trophoblast cells in culture, in which case it may be influenced by tissue context.

We are thankful for this important suggestion. We have now carried out CUT&Tag on differentiated cells and include the findings in the revised Figures 1B, 1D and S1C. Consistent with our observations using ATAC-seq data, we find that TE regulatory activity is diminished upon in vitro differentiation.

4. Are the IAP-initiated chimeric transcripts including new coding regions? If so, a Western Blot analysis of a few of the 27 candidates should be performed to prove this. Suv39h2 is a particularly interesting candidate where such protein analysis would be very informative.

We performed a search for ORFs in IAP-driven transcripts and identified a putative protein isoform of SUV39H2 that includes a portion of the IAP and that is larger than the canonical form by 28 kDa. However, by Western blot we see no major size shift in the main band when comparing placenta (where the IAP isoform predominates) with TSCs (where only the canonical form is expressed). We now include this in a new Supplementary Figure S5. To note is that in our hands the main SUV39H2 band runs at a lower molecular weight than expected (54 kDa), which could be due to buffer/gel conditions and/or expression of a shorter isoform (ENSMUSG00000026646, 46 kDa). But we are reassured that the antibody used has been validated in multiple human KO lines, as well as in at least one mouse knockdown model (PMID: 32698678).

5. A WB analysis should for sure be performed on the M. musculus and M. pahari placentas. The IHC staining is not interpretable as to whether or not SUV39H2 levels are reduced in M pahari.

We appreciate the reviewer's point, but the main hypothesis to be tested here was whether there was an obvious difference in the spatial distribution of SUV39H2, which we did not find. Any more subtle differences would be cell-type specific and would require complex cell sorting approaches before attempting a western blot. This would not affect our conclusion that, despite differences between species at the transcriptional level, this does not lead to an overt redistribution of SUV39H2 protein expression.

6. Could the authors please also provide more global proof of the CRISPRi success. The display of two candidate gene tracks is not very telling.

In the original submission we had included a subfamily-level analysis of IAP expression in the CRISPRi experiment (Figure S6A of the revised version). This shows a mild downregulation of IAP expression overall. Whilst an element-based analysis would be preferable due to potential caveats with subfamily-level analyses, very few TSC-expressed elements are sufficiently mappable to ensure a robust analysis, which is why we only showed two highly expressed loci where the effects of CRISPRi can be evaluated. Importantly, we observe effects on gene expression that, whilst mild, are non-random and support a role for IAPs in regulating the expression of nearby genes (Figure 4D).

Significance

General assessment:
Collectively, this is a carefully conducted study that needs to be bolstered by some few additional experiments, as suggested above. The discovery of changing patterns of repetitive element activity in differentiating trophoblast cells is important and intriguing, as it has direct impact on the evolutionary divergence of gene expression and, as a consequence, cell type differentiation, through the insertion of IAP and L1 elements close to placenta-expressed genes. This will be a major contributor and even driver of the barrier to inter-species hybridization that the placenta represents.

Advance:
Currently, the main TE elements known to drive placenta-specific gene expression are retrovirally derived LTR elements. Here, however, the authors show that the relevance of these elements diminishes in the mature placenta, and instead is taken over by a different class, the IAP elements. This is important, as many these elements retain the capacity for

retrotransposition, and thus actively contribute to ongoing evolutionary divergence of placental gene expression patterns that ultimately may drive speciation.

Audience:
The manuscript is not particularly easy to follow, even for the informed reader, and it appeals to a relatively specialized audience in the field of genome regulation coupled to evolutionary aspects of repetitive element insertion/transposition. The authors should be encouraged to spell out some aspects of their thought process throughout the study in some more detail, so not to "lose" the reader.

We have made multiple changes throughout the manuscript that we hope improve readability.

Reviewer #3

Evidence, reproducibility and clarity

The cis-regulatory roles of TEs in human/mouse TSCs have been extensively studied, yet in vivo studies on their roles in placenta tissue is largely absent. In this manuscript, Amante and colleagues compared the regulatory landscape of TEs across the trophoblast cell lines and placenta samples in human and mouse, and after revealing the shared and species-specific patterns (including some that are surprising), they further investigated the regulatory function of the murine-specific IAP retrotransposons in house mouse and other mouse strains. Specifically, it presents several findings regarding the shared and diverged function of TEs across: 1) in vivo vs. in vitro placental models, 2) human vs. mouse, 3) and different mouse strains. The writing is of good quality, the results are well visualized and interpreted, the conclusions are reasonable, and the novelty is high. It significantly extended previous studies from the same group as well as many other researchers. I think this manuscript should fit publication after a minor revision. Below I have a few comments:

1. In Fig. 1B, it seems the differences of TE enrichment between the same groups of samples (e.g., B6 TSC vs. GFP TSC) is also remarkable. Is this expectable? I am curious if such difference is robust, or it is just due to the TE sub-families with too few copies, whose enrichment can be influenced by just a couple of overlapping counts. The authors may double-check if possible.

This is an interesting hypothesis, but the main subfamilies that are H3K27ac-enriched in TSCs are quite abundant (e.g., 683 copies of RLTR13D5, 260 copies of RLTR13B3). We believe these are cell line-specific differences, possibly partly driven by genetics, since they were derived from

different mouse strains. Nonetheless, there is good agreement between the two lines with respect to the TE subfamilies that are enriched.

2. The authors demonstrate that the association of TEs to cis-regulatory elements is much weaker in the placenta of mouse relative to human, and in mouse the activation of TEs is indeed similar to most other tissues. And based on this observation, they propose that "Co-option of TEs as regulatory elements within the mature placenta may therefore not be as promiscuous across species as commonly thought" (page 4 paragraph 1). While this finding is quite interesting, how it is related to the popular hypothesis that "maternal-fetal conflict leads to the strong TE activation in placenta"? I am curious if the authors have any idea on this point.

It is indeed a fascinating topic. We would dispute that the conflict hypothesis leads to TE activation in the placenta, but rather that it creates selective pressures that drive their co-option. But this still requires for TEs to be available for co-option. What we suggest here is that TE co-option opportunities can be tightly constrained by transcriptional silencing mechanisms, even in the placenta. We added the following text to that section of the discussion: “Whilst maternal-fetal conflicts may create selective pressures for TE co-option in the placenta, epigenetic mechanisms can still act as gatekeepers and dictate the frequency of co-option events in this organ.”

3. For the highly active IAP subfamilies identified in mouse placenta, have the authors tried to identity the enriched motifs, which may be helpful for uncovering transcription factors responsible for their activation?

This is an interesting question, given the specific expression of IAP elements in the spongiotrophoblast. We now performed transcription factor motif analysis on subfamilies that are highly expressed in the placenta (IALTR1/2). We then filtered this list for motifs that are absent/mutated in lowly expressed IAP subfamilies (IAPLTR3/4) and whose associated transcription factor is highly expressed in spongiotrophoblast. In the revised manuscript we highlight our top candidate, MITF, which is a spongiotrophoblast-specific marker (Figure S3C).

4. In Fig. 3B, the IAPEY_LTR-adjacent Zfp229 gene is demonstrated, yet this gene is not mentioned at all in the main text. The authors may consider providing more details for this gene.

Unfortunately, nearly nothing is currently known about this zinc finger protein gene, but we did not feel that should prevent us from using it as a strong example of placenta-specific usage of

an IAP-derived promoter. Future work on this gene may indeed be triggered by highlighting this observation.

5. A few errors for the citations should be corrected. For example, the journal names are missed for ref56 and ref58 at page 22.

We have reviewed all our references and added missing information

6. A few typos should be corrected. For example, at page 11 line 2, "of" is missed between "presence this IAP-driven.

We have corrected this typo and made additional changes to the manuscript to improve readability.

Significance

Overall, this is an interesting and technically-sound study with substantial novelty, which significantly extends previous knowledge on TE function in placenta which largely relies on in vitro models.I believe this study will be attractive to the fields about TE function and placenta evolution.

October 16, 2026

RE: Life Science Alliance Manuscript #LSA-2026-03923

Miguel Branco
Queen Mary University of London
United Kingdom

Dear Dr. Branco,

Thank you for submitting your revised manuscript entitled "IAP retrotransposons contribute to the transcriptional diversity of the murine placenta". We returned this work to the original referees from Review Commons. As you will see, these experts are satisfied and recommend publication. We would be happy to publish your paper in Life Science Alliance pending final revisions necessary to meet our formatting guidelines.

MANUSCRIPT ORGANIZATION AND FORMATTING:

To avoid unnecessary delays in the acceptance and publication of your paper, please read the following information carefully. Full guidelines are available on our Instructions for Authors page, https://www.life-science-alliance.org/authors

Submission of a paper that does not conform to Life Science Alliance guidelines will delay the acceptance of your manuscript.

-Please add the X and Bluesky handles of your host institute/organization, as well as your own and/or one of the authors, in our system.
-Please upload your main manuscript text as an editable doc file.
-Please upload your main and supplementary figures as single files.
-Please add your main and supplementary figure and table legends to the main manuscript text after the references section.
-Please add a Running Title in our system.
-Please add a Summary Blurb/Alternate Abstract in our system.
-Please add a Category for your manuscript in our system.
-It is recommended to exclude figures from the manuscript text and upload them separately.
-Please rename "Competing interests" to "Conflict of Interest" and use this formulation: The authors declare that they have no conflict of interest.
-Please add a callout for Figure S7A to your main manuscript text.
-Please add an Author Contributions section to your main manuscript text and in our system.

We encourage our authors to provide original source data, particularly uncropped/-processed electrophoretic blots and spreadsheets for the main figures of the manuscript. If you would like to add source data, we would welcome one PDF/Excel-file per figure for this information. These files will be linked as supplementary "Source Data" files.

It is Life Science Alliance policy that if requested, original data images must be made available to the editors. Failure to provide original images upon request will result in unavoidable delays in publication. Please ensure that you have access to all original data images prior to final submission.

If you are planning a press release on your work, please inform us immediately to allow informing our production team and scheduling a release date.

We welcome submissions of potential cover images for the issue of LSA in which your work would appear. If you have high quality images associated with this work, please feel free to email these, with a caption, to the journal office.

LSA encourages authors to provide a 30-60 second video where the study is briefly explained. These videos will be appear embedded with the manuscript online at Life Science Alliance, and on social media to promote the published paper and authors (for examples, see https://docs.google.com/document/d/1-UWCfbE4pGcDdcgzcmiuJl2XMBJnxKYeqRvLLrLSo8s/edit?usp=sharing). Corresponding or first-authors are welcome to submit the video. Please submit only one video per manuscript. The video can be emailed to contact@life-science-alliance.org

FINAL FILES:

To upload the final version of your manuscript, please log in to your account: https://lsa.msubmit.net/cgi-bin/main.plex
You will be guided to complete the submission of your revised manuscript and to fill in all necessary information. Please get in

touch in case you do not know or remember your login name.

The following items are required for acceptance.

-- An editable version of the final text (.DOC or .DOCX) is needed for copyediting (no PDFs).

-- High-resolution figure, supplementary figure and video files uploaded as individual files: See our detailed guidelines for preparing your production-ready images, https://www.life-science-alliance.org/authors

-- Summary blurb (enter in submission system): A short text summarizing in a single sentence the study (max. 200 characters including spaces). This text is used in conjunction with the titles of papers, hence should be informative and complementary to the title. It should describe the context and significance of the findings for a general readership; it should be written in the present tense and refer to the work in the third person. Author names should not be mentioned.

The license to publish form must be signed before your manuscript can be sent to production. A link to the license to publish form will be available to the corresponding author only. Please take a moment to check your funder requirements.

Reviews, decision letters, and point-by-point responses associated with peer-review at Life Science Alliance will be published, alongside the manuscript. If you do want to opt out of having the reviewer reports and your point-by-point responses displayed, please let us know immediately.

Thank you for your attention to these final processing requirements. Please revise and format the manuscript and upload materials as soon as you are able.

Thank you for this interesting contribution to the literature. We look forward to publishing your paper in Life Science Alliance.

Sincerely,

Tim Fessenden
Scientific Editor
Life Science Alliance

Reviewer #1 (Comments to the Authors (Required)):

The authors have satisfactorily addressed my concerns.

Reviewer #2 (Comments to the Authors (Required)):

This is a carefully conducted study on the use of transposable elements (TEs) in the mature mouse placenta. Although TEs, and specifically LTR-derived long-terminal repeats, are well known to have important roles in stem cell-like trophoblast cells, a systematic analysis of the accessibility and use of such TE classes in the mature placenta has remained unknown.
The authors find that fully differentiated trophoblast cells use far fewer TE elements than trophoblast stem cells, and those they use are mainly derived from IAP elements. The authors perform various sophisticated analyses and incorporate many other publicly available datasets to show the imnpact of these IAPs on expression of nearby genes, as well as the identification of novel placenta-specific transcript isoforms. They perform a braod knockdown of IAP elements using CRISPRi, and also compare to distantly related mouse species so to assess the relevance of IAPs on placentation, placental divergence and evolution.
The study is of significant relevance to the field and also to a broader audience, not least as it challenges the current dogma that TE elements are particularly active in the placenta to drive tissue-specific transcript isoform expression. The study also contains a wealth of data that provides insightful advances into the subject from multiple different angles.
The authors have thoroughly addressed all comments previously raised in the Reviews Commons assessment process.
I have no further suggestions for additional amendments. I recommend acceptance of this manuscript.

Reviewer #3 (Comments to the Authors (Required)):

I appreciate the authors' efforts in revising the manuscript. All my concerns have been properly addressed.

October 20, 2026

RE: Life Science Alliance Manuscript #LSA-2026-03923R

Prof. Miguel R Branco
Queen Mary University of London
Blizard Institute
4 Newark Street
London E1 2AT
United Kingdom

Dear Dr. Branco,

Thank you for submitting your Research Article entitled "IAP retrotransposons contribute to the transcriptional diversity of the murine placenta". It is a pleasure to let you know that your manuscript is now accepted for publication in Life Science Alliance. Congratulations on this interesting work.

The final published version of your manuscript will be deposited by us to PubMed Central upon publication.

Your manuscript will now progress through copyediting and proofing. It is journal policy that authors provide original data upon request.

Your article will publish open access upon publication under a CC-BY license.

Reviews, decision letters, and point-by-point responses associated with peer-review at Life Science Alliance will be published, alongside the manuscript. If you do want to opt out of having the reviewer reports and your point-by-point responses displayed, please let us know immediately.

IMPORTANT: If you will be unreachable at any time, please provide us with the email address of an alternate author. Failure to respond to routine queries may lead to unavoidable delays in publication.

Scheduling details will be available from our production department. You will receive proofs shortly before the publication date. Only essential corrections can be made at the proof stage so if there are any minor final changes you wish to make to the manuscript, please let the journal office know now.

DISTRIBUTION OF MATERIALS:
Authors are required to distribute freely any materials used in experiments published in Life Science Alliance. Authors are encouraged to deposit materials used in their studies to the appropriate repositories for distribution to researchers.

You can contact the journal office with any questions, contact@life-science-alliance.org

Again, congratulations on a very nice paper. I hope you found the review process to be constructive and are pleased with how the manuscript was handled editorially. We look forward to future exciting submissions from your lab.

Sincerely,

Tim Fessenden
Scientific Editor
Life Science Alliance
